# Transcriptome Analysis and Gene Expression Profiling of the Peanut Small Seed Mutant Identified Genes Involved in Seed Size Control

**DOI:** 10.3390/ijms23179726

**Published:** 2022-08-27

**Authors:** Fengdan Guo, Xiujin Zhu, Chuanzhi Zhao, Shuzhen Zhao, Jiaowen Pan, Yanxiu Zhao, Xingjun Wang, Lei Hou

**Affiliations:** 1 Institute of Crop Germplasm Resources, Shandong Academy of Agricultural Sciences, Shandong Provincial Key Laboratory of Crop Genetic Improvement, Ecology and Physiology, Jinan 250100, China; 2 Institute of Chinese Medicine Resources, Shandong Academy of Chinese Medicine, Jinan 250014, China; 3 College of Life Sciences, Shandong Normal University, Jinan 250014, China

**Keywords:** *Arachis hypogaea* L., seed size, small seed mutant, RNA-seq

## Abstract

Seed size is a key factor affecting crop yield and a major agronomic trait concerned in peanut (*Arachis hypogaea* L.) breeding. However, little is known about the regulation mechanism of peanut seed size. In the present study, a peanut *small seed mutant1* (*ssm1*) was identified through irradiating peanut cultivar Luhua11 (LH11) using ^60^Coγ ray. Since the globular embryo stage, the embryo size of *ssm1* was significantly smaller than that of LH11. The dry seed weight of *ssm1* was only 39.69% of the wild type LH14. The seeds were wrinkled with darker seed coat. The oil content of *ssm1* seeds were also decreased significantly. Seeds of *ssm1* and LH11 were sampled 10, 20, and 40 days after pegging (DAP) and were used for RNA-seq. The results revealed that genes involved in plant hormones and several transcription factors related to seed development were differentially expressed at all three stages, especially at DAP10 and DAP20. Genes of fatty acid biosynthesis and late embryogenesis abundant protein were significantly decreased to compare with LH11. Interestingly, the gene profiling data suggested that *PKp2* and/or *LEC1* could be the key candidate genes leading to the small seed phenotype of the mutant. Our results provide valuable clues for further understanding the mechanisms underlying seed size control in peanut.

## 1. Introduction

Peanut (*Arachis hypogaea* L.), an important oil crop, is widely grown around the world. Peanut seeds are rich in oil, protein, and many other nutritional components, such as resveratrol, isoflavones, vitamin, and biotin [1]. Seed size is a key factor affecting peanut yield and quality. Studies in Arabidopsis (*Arabidopsis thaliana*), rice (*Oryza sativa* L.), and other species have identified several signaling pathways and key genes that control seed size [2,3,4,5,6]. In peanut, there are studies that reported the identification of QTLs related to seed size through different approaches [4,5,6]. However, the molecular mechanism and regulatory networks underlying peanut seed size is still unclear.

Seed size is determined by coordinated growth of maternal integument, endosperm, and embryo. Several signaling pathways, such as the G-protein signaling pathway, mitogen-activated protein kinase (MAPK) signaling pathway, ubiquitin-proteasome signaling pathway, and HAIKU (IKU) pathway, have been identified to regulate seed size [7]. *IKU1*, *IKU2*, *MINI3*, and the upstream regulator *Short Hypocotyl under Blue 1* (*SHB1*) play positive roles and increase seed size through promoting the growth of endosperm [8,9,10]. The legume seed development includes three typical stages: embryo morphogenesis phase, storage phase, and desiccation phase [11,12]. ABA play vital roles in seed development. During early embryo development, ABA promotes embryo growth [13]. The mutation of key genes in ABA biosynthesis and signaling transduction pathways led to defect of early embryogenesis in Arabidopsis [14]. During the seed maturation, ABA appear to promote accumulation of storage reserves [15]. Auxin and cytokinin also play critical roles in plant embryogenesis. *Auxin responsive factor 2* (*ARF2*) plays negative roles in seed development and the mutant *arf2* seeds are larger than the wild type control [16]. The mutation of cytokinin receptor genes could also generate larger seeds in Arabidopsis [17]. However, mutation of genes in brassinosteroid (BR) signaling pathway led to dwarf plant and small seeds [18,19,20,21,22].

Several transcriptional regulators are also involved in the regulation of seed size, such as *ABI3*, *FUS3*, *LEC1*, and *LEC2* [15,23]. *LEC1* could regulate gene expression during seed maturation by affecting the expression levels of *ABI3*, *FUS3*, and *WRI1* [24,25]. *WRI1* encodes a putative AP2/EREBP transcription factor. The mutant *wri1-1* in Arabidopsis and the knockout line of *TaWRI1L2* in wheat both produced small, wrinkled seeds with reduced oil accumulation [26,27]. Plastidial pyruvate kinase (PKp) is the key rate-limiting enzyme in glycolysis and provides acetyl-CoA for de novo synthesis of fatty acids [28]. The expression of *PKp* was directly or indirectly regulated by *LEC1* and *WRI1* [25,29]. The mutation of *pkp1* and *pkp2* in Arabidopsis led to defect in embryo elongation and produced small, wrinkled seeds with only 30%~50% fatty acid content of the WT seeds [30]. AP2/EREBP is a major transcription factor family that determines floral organ characteristics. Loss of function or suppression of *AP2* gene expression in Arabidopsis resulted in enlarged integument cells and seeds [31]. The mutation of *ANT*, an *AP2-like* gene, resulted in small seeds [32]. WRKY transcription factors play important roles during seed development and eventually affect seed size. In Arabidopsis, the mutation of *MINI3* which encodes a WRKY10 produced seeds with significantly reduced size [9]. The mutation of *TTG2*, another WRKY family member, resulted in small seed due to the restricted endosperm growth and integument cell elongation [33]. Previous studies demonstrated that B-class MADS-box transcription factors are negative regulators on seed size. The loss of function of these genes led to large seeds in Arabidopsis or long grains in rice [34,35,36,37].

To date, no gene that regulates seed size has been identified in peanut yet. To understand the molecular regulation mechanism of peanut seed size and search for the gene controlling peanut yield and quality, we identified a *small seed mutant1* (*ssm1*) through irradiating peanut cultivar Luhua11 (LH11) using ^60^Coγ ray. In the present study, the phenotypic characteristics, endogenous hormone levels, and seed transcriptome of *ssm1* were investigated. The results provided valuable information for candidate genes identification and understanding the molecular mechanism underlying seed size regulation in peanut.

## 2. Results

### 2.1. SSM1 Mutant Acquisition, Genetic and Phenotypic Analyses

**Figure 1:**
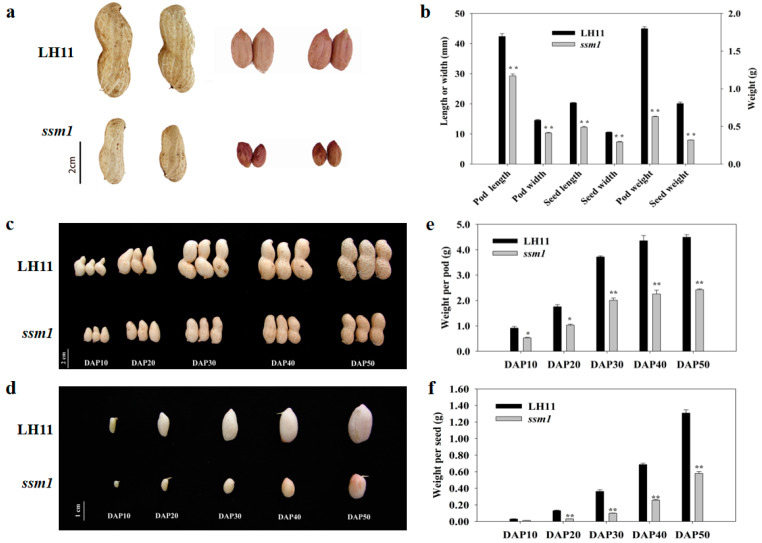
Comparison of pods and seeds between mutant *ssm1* and LH11. (**a**) Mature pods and seeds of *ssm1* and LH11; (**b**) Mature pod and seed length, width, and weight of *ssm1* and LH11; (**c**) The pods of *ssm1* and LH11 at different developmental stages; (**d**) Seeds of *ssm1* and LH11 at different developmental stages; (**e**) Pod weight of *ssm1* and LH11 at different developmental stages; (**f**) Seed weight of *ssm1* and LH11 at different developmental stages. DAP10, 10 days after pegging; DAP20, 20 days after pegging; DAP30, 30 days after pegging; DAP40, 40 days after pegging; DAP50, 50 days after pegging. Three biological replicates were used for statistical analysis (*t*-test; * *p* < 0.05, ** *p* < 0.01). Values in (**b**,**e**,**f**) represent means ± SE (*n* = 3).

A peanut mutant with small pod and small seed was named *ssm1*. It was identified from a mutant population induced by ^60^Co γ-ray radiation using cultivar Luhua 11 (LH11). Compared with LH11, the pod and seed of *ssm1* were significantly smaller, and the seed coat was wrinkled with darker color. At maturity, the length, width, and weight of the pod of *ssm1* decreased significantly. The pod and seed dry weight of *ssm1* were only 35.04% and 39.69% of the control LH11, respectively (Figure 1a,b). At different development stages, the pod and seed weight of *ssm1* were all much smaller than that of LH11. For example, the fresh weight of *ssm1* seeds was reduced by 57.01%, 76.20%, 72.71%, 62.58%, and 55.67% compared with LH11 at DAP10 to DAP50 stages, respectively (Figure 1c–f). The oil content of dry seeds of *ssm1* was 46.7%, which was 13.53% less than that of LH11. The protein content of *ssm1* was 22.60%, which was similar to that of LH11.

The germination rate and germination potential of *ssm1* were only 86.44% and 77.97% of LH11. The peanut seed development is not synchronized and that makes it difficult for investigate the phenotype of the seed in F_2_ population. To overcome this difficulty, we modified the phenotyping method in this study. We planted each F_2_ plant and harvested a number of F_3_ seeds, then the seed size of F_3_ seeds was investigated. If the F_3_ seeds in one plant are all small in size, we considered the genotype of the corresponding F_2_ seed/plant as a small seed homozygote mutant. In generation of Tif × *ssm1* cross, the F_2_ large seeds to small seeds ratio (L/S) is 4.52:1 (1212 large seed plants and 268 small seed plants). The low germination rate of the small seeds (77.97%) could be the major reason that caused the deviation of L/S from 3:1. The mutant is inherited stably, and we speculated that small seed trait is controlled by a single gene.

### 2.2. Comparison of Early Embryo Development

**Figure 2:**
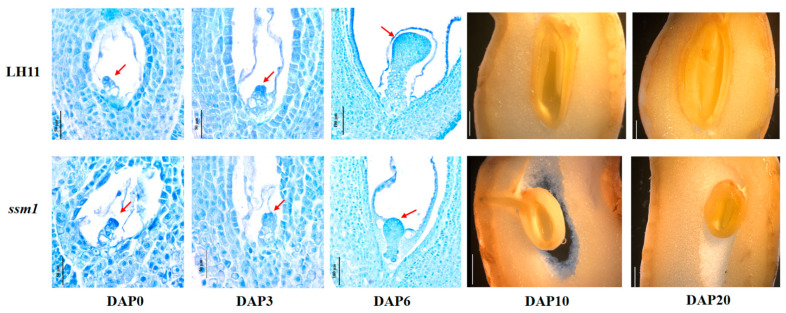
Early embryonic development differences between *ssm1* and LH11. DAP0, 0 day after pegging; DAP3, 3 days after pegging; DAP6, 6 days after pegging; DAP10, 10 days after pegging; DAP20, 20 days after pegging. Red arrows represent embryos.

In order to investigate the difference between the embryo development of *ssm1* and LH11, embryos at different developmental stages were observed (Figure 2). Tissue sections showed that the trend of embryo development of *ssm1* was consistent with LH11, but the size of embryo was smaller than LH11 significantly. The embryo at DAP0 was in the rod-like pro-embryo stage, and there was no significant difference between *ssm1* and LH11. At DAP3, the embryo size was similar to that of DAP0 embryo in both *ssm1* and LH11. At DAP6, the embryo of *ssm1* and LH11 were both at the globular stage, but the embryo size of *ssm1* was much smaller than LH11. At DAP10 and DAP20, the embryos of *ssm1* were significantly smaller than that of LH11. A space between the ovule and ovary wall was observed in *ssm1* due to the inconsistent development of the ovule and ovary, which was not occurred in LH11. The results indicated that *ssm1* was different from LH11 during early embryo development from the globular stage, and the embryo was smaller than LH11.

### 2.3. Changes of Endogenous Hormone Content in SSM1

**Figure 3:**
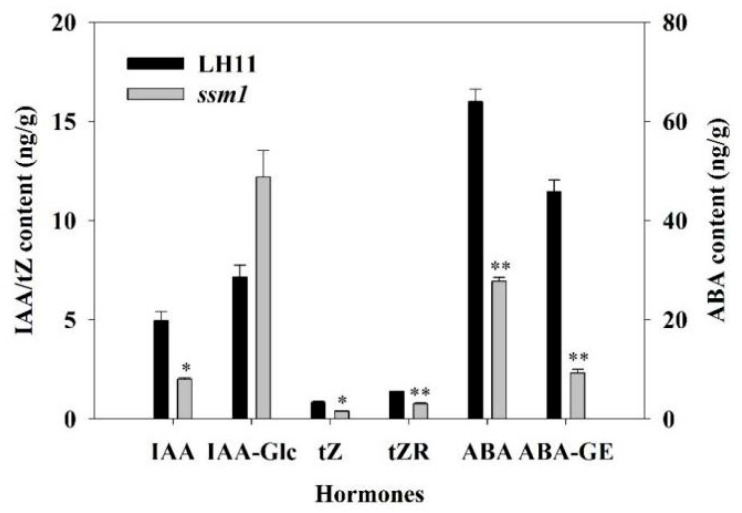
Hormone content in seeds of *ssm1* and LH11 at DAP20. IAA: Indole-3-acetic acid; IAA-Glc: 1-O-indol-3-ylacetylglucose; tZ: trans-Zeatin; tZR: trans-Zeatin Riboside; ABA: Abscisic acid; ABA-GE: ABA-glucosylester. Three biological replicates were used for statistical analysis (*t*-test; * *p* < 0.05, ** *p* < 0.01). Values represent means ± SE (*n* = 3).

Endogenous auxin, cytokinin, and abscisic acid (ABA) concentrations were determined in *ssm1* and LH11 seeds of DAP20 (Figure 3). Indole-3-acetic acid (IAA) in *ssm1* was only 40.57% of that in LH11, while the content of a storage form of 1-O-indole-3-yl acetyl glucose (IAA-Glc) in *ssm1* was much higher than that in LH11. Trans-zeatin (tZ) and trans-zeatin riboside (tZR) are the main active cytokinins, and their content in *ssm1* were about 47.01% and 54.80% of that in LH11, respectively. The content of ABA and ABA-glucosylester (ABA-GE) in *ssm1* seeds were significantly reduced at only 43.43% and 20.16% of that in LH11, respectively.

### 2.4. Transcriptome Differences between SSM1 and LH11 during Seed Development

**Figure 4:**
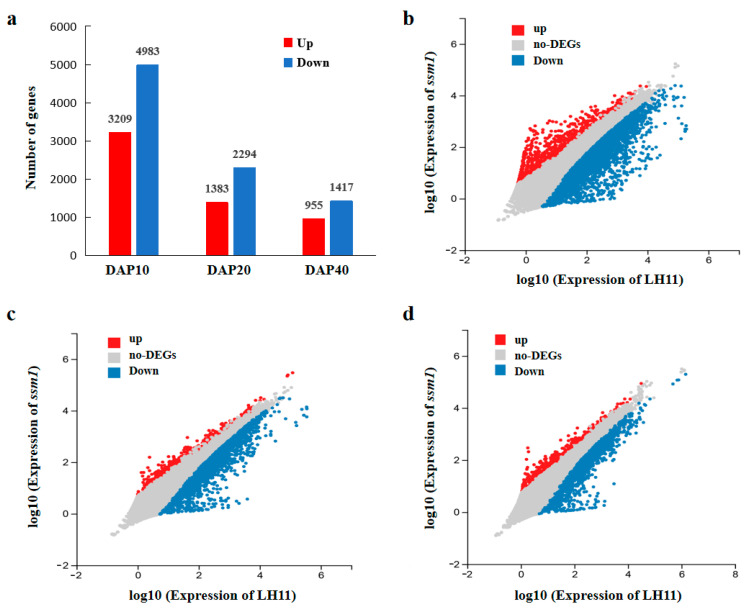
Comparison of DGEs between *ssm1* and LH11 seeds. (**a**) Numbers of DGEs in seeds between *ssm1* and LH11 at DAP10, DAP20, and DAP40 three stages; (**b**) Scatter diagram of DEGs in seeds at DAP10 between *ssm1* and LH11; (**c**) Scatter diagram of DEGs in seeds at DAP20 between *ssm1* and LH11; (**d**) Scatter diagram of DEGs in seeds at DAP40 between *ssm1* and LH11. Both the X and Y axes represent log10 values of gene expression.

In order to understand the changes at the transcription level, RNA-seq of seeds in different developmental stages were carried out. A total of 390.36 million raw reads were generated in this study (Supplementary Table S1). From each sample, we got 21.63 million clean reads on average. The average mapping ratio of clean reads to the reference genome and reference genes were 95.87% and 81.75%, respectively. The results showed that the reads were evenly distributed in each region of the transcripts, indicating that the sequencing data could accurately reflect the gene expression level. In total, 8192, 3677, and 2372 differentially expressed genes (DEGs) were identified in DAP10, DAP20, and DAP40 seeds between *ssm1* and LH11, respectively (Figure 4; Supplementary Table S2). At DAP10, the number of DEGs was the largest, with 3209 and 4983 up-regulated and down-regulated genes in *ssm1*, respectively.

**Figure 5:**
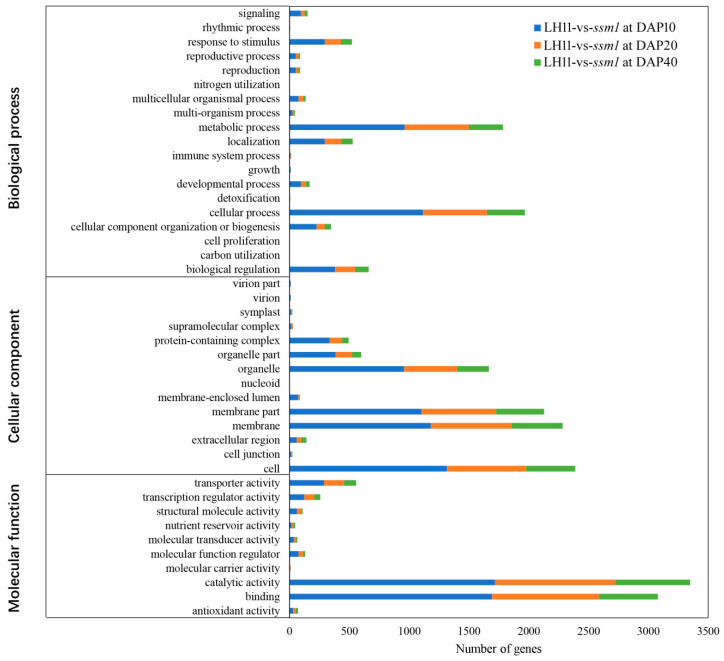
GO classification analysis of DEGs in seeds of *ssm1* and LH11. The *X*-axis represents the number of genes annotated into the GO terms, and the *Y*-axis represents the functional classification of GO.

To gain insights into the functional categories of the DEGs between *ssm1* and LH11, Gene Ontology (GO) enrichment analysis were conducted. The DEGs were classified into three main categories: biological process, cellular component, and molecular function (Figure 5). Cellular process and metabolic process were the top two terms in the biological process. In the cellular component category, DEGs were mainly distributed in terms of cell, membrane, membrane part, and organelle. The most abundant terms in the molecular function were catalytic activity and binding, which indicated that the mutants had a high degree of gene changes involved in binding and catalytic processes. At DAP10, 505 and 329 genes were annotated as the nucleus and DNA binding terms, respectively, most of which were transcription factors. At DAP20, the DEGs were enriched significantly in the synthesis and metabolism of nutriments, including fatty acid, carboxylic acid, lipid, and organic acid, etc. At DAP40, the enriched terms included carbohydrate, polysaccharide metabolic process, monolayer-surrounded lipid storage body, and nutrient reservoir activity, which were involved in nutrient accumulation.

**Figure 6:**
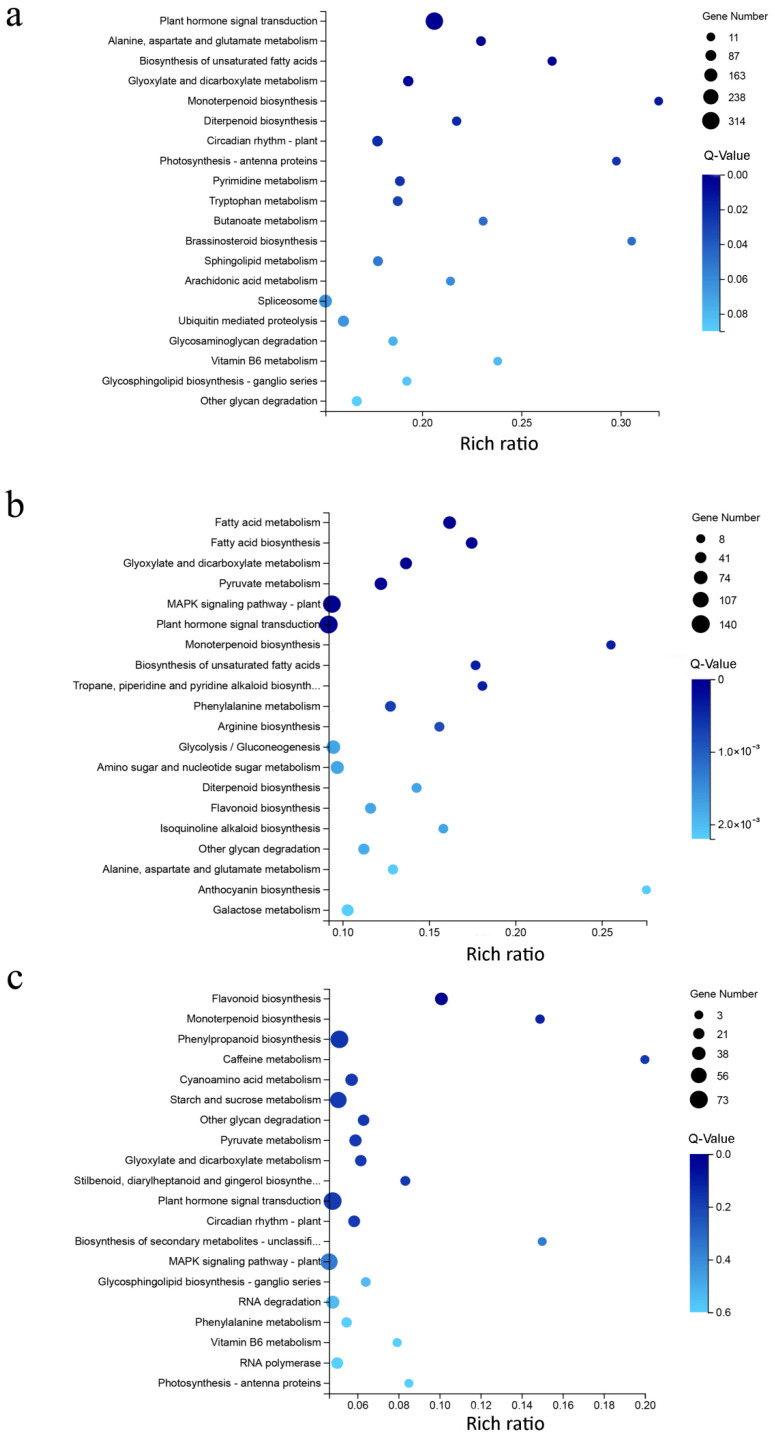
Bubble diagram of top 20 enriched KEGG pathways of DEGs in seeds of *ssm1* and LH11 at three stages. (**a**) DAP10; (**b**) DAP20; (**c**) DAP40. *X*-axis represents the rich ratio, which means the ratio of selected gene number annotated to a particular item to the total number of genes in this item in one species. The calculating formula is Rich Ratio = Term Candidate Gene Num/Term Gene Num. *Y*-axis represents KEGG Pathway. The size of the bubbles indicates the number of genes annotated to a KEGG Pathway. The color represents Q-value of enrichment. The deeper the color, the smaller the Q-value.

Kyoto Encyclopedia of Genes and Genomes (KEGG) pathway analysis classified the DEGs into 132, 132, and 127 metabolic pathways at DAP10, DAP20, and DAP40, respectively (Supplementary Table S3). At DAP10, plant hormone signal transduction pathway was the most abundant pathway. Diterpenoid biosynthesis, tryptophan metabolism, and brassinosteroid biosynthesis were observed in the top 20 enriched pathways (Figure 6), which indicated that hormones were changed significantly in *ssm1*. At DAP20, fatty acid synthesis and metabolism was the most abundant pathway, and sugar metabolism related pathways such as glycolysis/gluconeogenesis and some amino acids metabolism were also enriched. These results suggested that the pathways related to nutrient accumulation were the main difference between *ssm1* and LH11. At DAP40, flavonoid biosynthesis was the most enriched pathway, and metabolism pathways of starch, sucrose, and some amino acids were also enriched. In addition, plant hormone signal transduction and mitogen-activated protein kinase (MAPK) signaling pathway were also enriched at DAP20 and DAP40.

### 2.5. Genes Expression Changes in Hormone Biosynthesis and Signal Transduction

Plant hormones play important roles in the regulation of seed development. RNA-seq results showed that the expression of auxin synthesis-related genes was significantly different in *ssm1* (Supplementary Table S4). Tryptophan aminotransferase of Arabidopsis 1/tryptophan aminotransferase related (TAA1/TARs) and YUCCA are the key enzymes in the indole-3-pyruvic acid pathway of auxin biosynthesis. One TAA1/TAR gene showed decreased expression at DAP10 in *ssm1*, which was 41% of the expression level of LH11. The expression of six YUCCA family genes were up-regulated at DAP10 and DAP20. Amidase (AMI) catalyzed indole-3-acetamide (IAM) to generate IAA, and the expression levels of two AMI1 genes were significantly reduced. ALDH is the key enzyme catalyzing indole-3-acetaldehyde (IAAld) to IAA. The expression level of six ALDH genes in DAP10 and DAP20 of *ssm1* seeds were down-regulated to compare with LH11, which was consistent with the decreased IAA content in *ssm1* seeds. UDP-glycosyltransferases (UGTs) are responsible for catalyzing auxin glycosylation. At DAP10, the expression of four UGTs decreased in *ssm1*, while the expression of four other UGTs increased significantly at DAP20, which was consistent with the high content of IAA-Glc in *ssm1* seeds at DAP20. All five SAUR genes were down-regulated in *ssm1* seeds at DAP10 and DAP20. The expression levels of the majority auxin response factors (ARF) genes were decreased more than one-fold in DAP10 and DAP20. Two auxin receptors TIR1 genes were also down-regulated in *ssm1*. Except for YUCCAs, the expression changes of these genes were consistent with the decreased IAA content in *ssm1* seeds at DAP20, while these auxin synthesis and response genes were not changed obviously at DAP40.

DEGs in ABA synthesis and signal transduction pathways between *ssm1* and LH11 seeds at DAP10, DAP20, and DAP40 showed an overall trend of down-regulation. Most ABA biosynthesis genes were significantly decreased in *ssm1* seeds to compare with LH11, for example, beta-carotene 3-hydroxylase (BCH1), 9-cis-epoxycarotenoid dioxygenase (NCED), and ABA deficient 2/short-chain dehydrogenase reductase 1(ABA2/SDR). The CYP707A2 genes involved in ABA degradation were down-regulated at DAP10 and DAP40, but up-regulated in DAP20. ABA glucosyltransferase is responsible for catalyzing ABA glycosylation, and the expression level of four coding genes in *ssm1* were all down-regulated at DAP10 and DAP20. The expression trends of these genes were consistent with the reduced trends of ABA-GE content at DAP20. The expression levels of the important transcription factor ABI5 in ABA signaling pathway were lower in *ssm1* seeds than those in LH11, indicating that ABA signal transduction was also affected in *ssm1* seeds.

Cytokinins (CKs) play key roles in plant growth and development. The levels of endogenous CKs in plants are influenced by isopentenyl transferases (IPT), cytochrome P450 monooxygenase (CYP735A), cytokinin oxidase/dehydrogenase (CKX), and other biosynthetic and degrading enzymes. The expression levels of three CYP735A genes up-regulated in *ssm1*; however, the expression level of most of the CKX genes, which degraded CKs, were increased in *ssm1* seeds at three developmental stages. The increased expression of CKX genes in *ssm1* presumably was the cause of the decrease of CKs in *ssm1*. Meanwhile, the expression of receptor CRE1 genes in CKs signal transduction were down-regulated in *ssm1* seeds.

The expression levels of biosynthesis and signal transduction genes of brassinolide (BR) and gibberellin (GA) also changed in *ssm1* seeds. The expression levels of most genes that catalyze BRs biosynthesis in *ssm1* seeds at DAP10 and DAP20 were significantly lower than those of LH11, including DWF4/CYP90B1, CPD/CYP90A1, CYP90C1/D1, and Dwarf/CYP85A1. The expression of BES1/BZR1, a key transcription factor in BRs signal transduction, was decreased in *ssm1* seeds at all three developmental stages. The key enzyme genes KAO and KO that catalyze GAs biosynthesis were down-regulated in *ssm1* seeds at DAP10 and DAP20, and the expression of some GA20ox were down-regulated; however, the expressions of other GA20ox genes with low expression background were up-regulated. DELLA protein and GA receptor GID1 genes were down-regulated in *ssm1* seeds at DAP10 and DAP20. These results indicated that BR and GA biosynthesis and signaling were affected in *ssm1* seeds.

### 2.6. Genes Expression Changes of Storage Materials

**Figure 7:**
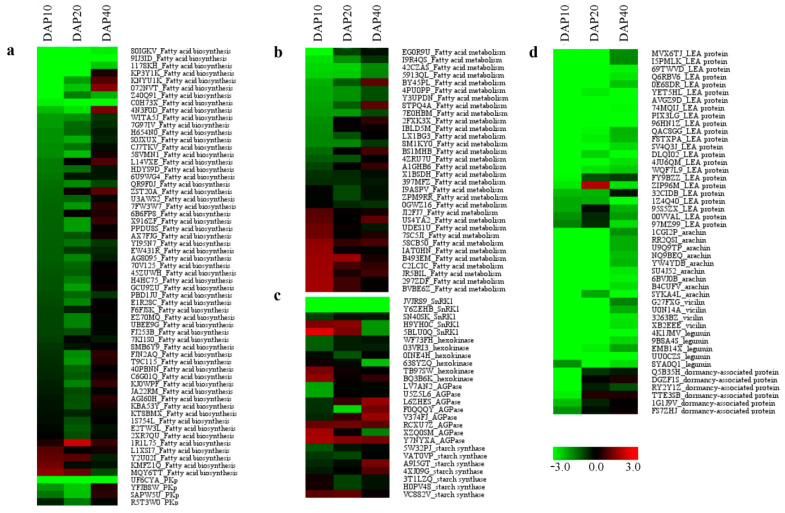
The expression pattern of storage reserves-related genes in seeds between *ssm1* and LH11. Different expressions of the related genes of fatty acid biosynthesis (**a**), fatty acid metabolism (**b**), starch synthesis (**c**), and late embryogenesis abundant proteins (**d**) in seeds between *ssm1* and LH11. Scale bar is located at upside with log_2_ ratio value varying from green to red.

The genes encoding key enzymes involved in fatty acid synthesis and metabolism including acetyl-CoA carboxylase (ACCase), acyl carrier protein 1 (ACP1), beta-ketoacyl-ACP synthase (KAS), and diacylglycerol acyltransferase (DGAT) were significantly down-regulated in *ssm1* seeds to compare with LH11 at three developmental stages (Figure 7a,b). Plastic pyruvate kinase (PKp) and fructose-1,6-bisphosphatase (FBP) are the key enzymes in the glycolysis pathway, which provides the substrate acetyl-coA for the de novo synthesis of fatty acids. The expression of all *PKp* and *FBP* genes were significantly decreased in *ssm1* seeds (Figure 7a). Late embryogenesis abundant proteins (LEA) are abundantly accumulated in seeds during late embryonic development. Arachin, vicilin, legumin, and dormancy-associated proteins are also important storage proteins in seeds. The expression of all these genes encoding these proteins in *ssm1* seeds at DAP10, DAP20, and DAP40 were significantly decreased compared to LH11 (Figure 7d).

### 2.7. The Expression Changes of Transcription Factor Genes

RNA-seq analysis showed that the expression level of LEC1, AP2/EREBP, FUS3, ABI3, and GRF transcription factors encoding genes were significantly down-regulated in *ssm1* of at DAP10 and DAP20 to compare with those of LH11, while the expression level of SPL and MADS-Box transcription factor genes were up-regulated in *ssm1* (Supplementary Table S5). The IKU pathway positively regulates seed development by regulating endosperm growth. The expression level of IKU1 and IKU2 genes in *ssm1* seeds at DAP10 were significantly lower than those of LH11, while the expression of some IKU2 genes were up-regulated in *ssm1* seeds at DAP40. The expression of RAV1, which negatively control IKU2 expression in Arabidopsis, were up-regulated at DAP10 and DAP20 and down-regulated at DAP40 in *ssm1* seeds.

### 2.8. Verification of DEGs Using qRT-PCR

**Figure 8:**
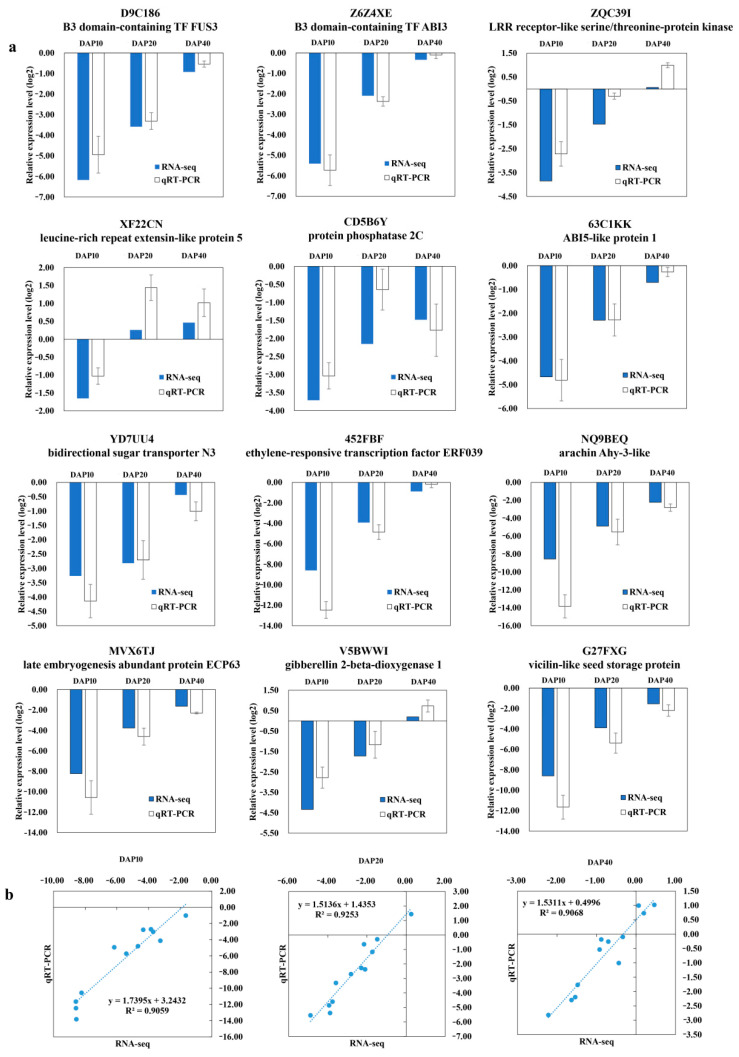
Verification of DEGs by qRT-PCR in seeds. (**a**) Verification of 12 DEGs by qRT-PCR in seedss. DAP10, DAP20, and DAP40 were the relative gene expression levels of *ssm1* and LH11 seeds at DAP10, DAP20, and DAP40, respectively. (**b**) Pearson’s correlation of gene expression ratios between RNA-seq and qRT-PCR results.

To validate the RNA-seq results, 12 DEGs were randomly selected for qRT-PCR analysis. These genes include transcription factor genes, storage protein genes, and genes related to hormone biosynthesis and signal transduction. The qRT-PCR results of these genes were found in accordance with the relative expression levels obtained from RNA-seq. The correlation coefficients between qRT-PCR and RNA-seq data at DAP10, DAP 20, and DAP40 were 0.9059, 0.9253, and 0.9068, respectively (Figure 8 and Supplementary Table S6).

## 3. Discussion

The mechanism of seed size regulation has been extensively studied in Arabidopsis and many crops. However, little is known about the regulation mechanism of peanut seed size. In this study, *ssm1* had decreased oil content and the seed size was significantly smaller than that of LH11. The differences of ovule and embryo size between *ssm1* and LH11 become more obvious at DAP10 and DAP20 periods, and the development of seed integument was restricted.

Phytohormones play essential roles in seed development. The RNA-seq data presented in this study showed that genes involved in hormone signaling pathway in *ssm1* seeds changed greatly compared with LH11. Most of the catalyzing enzyme genes in ABA or BRs biosynthesis pathways and the regulatory transcription factor genes were down-regulated in *ssm1* seeds at three developmental stages, especially in DAP10 and DAP20. These results suggested that ABA and BRs biosynthesis and signaling might decrease in *ssm1* seeds at early developmental stage. Our results showed that ABA and ABA-GE content in *ssm1* seeds of DAP20 was significantly lower than LH11. Previous studies demonstrated that ABA play critical roles during early seed development through regulating embryonic cell division and endosperm cellularization. ABA-deficient mutant *aba2-1* exhibited delayed embryogenesis compared with the wild type [14]. BRs regulate seed size and shape in Arabidopsis. BR-deficient affect seed cavity and endosperm development and eventually the seed size. The embryos of BR deficient mutant *det2* were smaller than the wild type control due to the reduced cell size and cell number [20]. SHB1-MINI3-IKU2 cascade was a critical regulator promoting endosperm and embryo development and in Arabidopsis. The mutation of each gene of this cascade resulted in small seeds [9,10,38]. RAV1 is the negative player during early seed development by repressing *IKU2* expression. *IKU2* expression was significantly inhibited in overexpressing *RAV1* transgenic plant which produced small seeds [39]. Both ABA and BR inhibited the expression of *RAV1* [40,41]. Consistent with these results, in *ssm1*, the expression of *RAV1* was up-regulated at DAP10 and DAP20, then decreased sharply at DAP40 compared with LH11. The expression profile of *IKU2* was opposite to *RAV1*. Therefore, the reduced levels of ABA and BRs and the elevated expression of *RAV1* might have affected the expression of the core components that regulate embryo and endosperm development during the early stage of seed development in *ssm1*.

Auxin plays key roles during seed development, including embryo pattern formation, cell division, cell expansion, and embryo structure determination and seed size [42,43,44]. Ovule fertilization triggered auxin production, and then functioning in both the maternal tissue and the embryo to drive seed development [44]. In this study, IAA decreased in *ssm1* seeds while IAA-Glc increased. IAA sugar conjugates is an inactive form of auxin; UDP-glycosyltransferases (UGTs) catalyze the conjugation of sugar moieties and IAA [45,46,47]. Correspondingly, the expression of four *UGT* genes up-regulated in *ssm1* seeds at DAP20. *Small auxin up RNA* (*SAUR*) genes are the largest auxin responsive gene family, regulating auxin mediated cell elongation [48]. All *SAUR* genes, most *ARFs*, and the auxin receptor *TIR1* were down-regulated in *ssm1* seeds at DAP10 and DAP20. It was deduced that the weak IAA biosynthesis and signaling might affect the development of *ssm1* embryo and integuments, which results in small seed.

PKp2 catalyzes phosphoenolpyruvate to pyruvate in glycolysis and provides substrate for de novo fatty acid biosynthesis. The *PKp2* loss of function affected seed development and seed size. In Arabidopsis *pkp2*, *pkp1pkp2* mutants, the seed oil content reduced 50%~70% [30]. In *wri1* mutant with low *PKp2* expression, seed oil content decreased 80% [49].

The oil content of *ssm1* dry seeds reduced 13.53%. The oleic acid (C18:1) and linoleic acid (C18:2) contents decreased, while the linolenic acid (C18:3) and erucic acid (C22:1) contents increased in *ssm1* seed. The variation trend of fatty acid compositions is similar to that in *pkp2*, *pkp1pkp2* mutants. The expression of *PKp2* was hardly detected in *ssm1* at DAP10 and DAP20. In the promoter of *AhPKp2* gene, several hormone response elements including ABA, MeJA, GA, and auxin were identified. The reduced level of ABA and auxin in *ssm1* seeds may be the key reason for the reduced expression of *PKp2*. On the other hand, the functional defects of *PKp2* may affect the synthesis of plant hormones. Previous studies showed that loss of function of some enzymes involved in seed development usually affected multiple pathways, including the biosynthesis of plant hormones, overall metabolism, and composition of the storage materials [50,51,52]. Besides entering the fatty acid and starch synthesis pathways, the substrate phosphoenolpyruvate and the product pyruvate of PKp could also enter a variety of metabolic pathways, such as amino acid metabolism, terpene metabolism, and sterol metabolism. These pathways could provide precursors for the biosynthesis of IAA, cytokinins, gibberellin, brassinosteroids, and abscisic acid [53].

Several transcription factors have been proved playing key roles in seed size control. ABA play a key role in embryo maturation by inducing the expression of storage-related genes. Studies have shown that de-regulation of ABA impaired accumulation of storage materials in seed, which is closely related to the transcription factors *LEC1*, *WRI1*, *ABI3*, and *FUS3* [24,54,55]. LEC1 act as key regulators to coordinate the expression of fatty acid biosynthetic genes; overexpression of *LEC1* increased expression of over 58% of genes in fatty acid biosynthesis pathway. Moreover, genes of *Sucrose Synthase 2* (*SUS2*) and *PKp2* were also up-regulated upon *LEC1* overexpression. Genetic analysis indicates that the *LEC1* function is partially dependent on *WRI1*, *ABI3*, and *FUS3* in the regulation of fatty acid biosynthesis [55]. Consistent with these results, our RNA-seq results showed that the expression of *LEC1*, *FUS3*, *ABI3*, and many genes involved in fatty acid biosynthesis and metabolism were significantly decreased in DAP10 and DAP20 seeds of *ssm1*.

**Figure 9:**
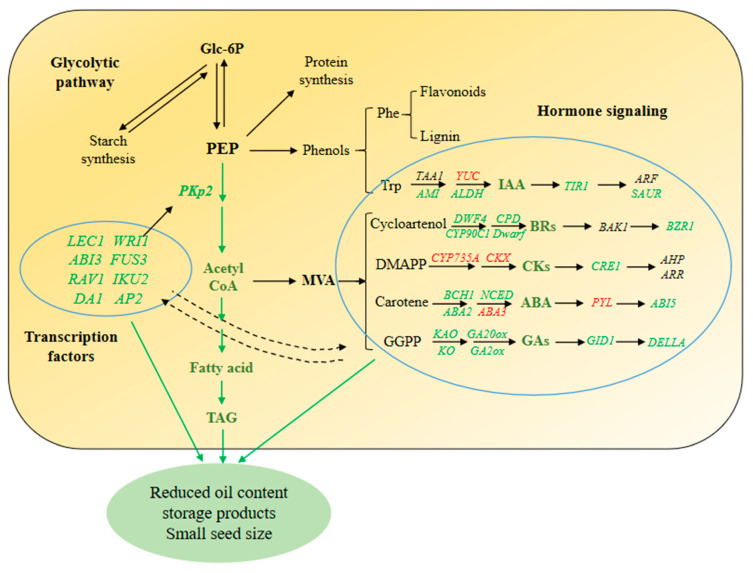
The regulatory model underlying small-seed phenotype in *ssm1* mutant. Glc-6P, D-Glucose 1-phosphate; PEP, Phosphoenol pyruvate; TAG, Triglyceride; Phe, Phenylalanine; Trp, Tyrosine; MVA, Mevalonic acid; DMAPP, Dimethylallyl diphosphate; GGPP, Geranylgeranyl diphosphate. Genes, metabolites, or arrows with green color indicate down-regulated expressions or reduced contents; those with red color indicate up-regulated expressions or increased contents.

Based on these results, we speculated that *PKp2* and *LEC1* could be the key candidate genes leading to the small seed phenotype of the mutant. Together, we hypothesized that the hormone signaling pathways and transcription factors cooperatively modulate seed development and storage accumulation (Figure 9). How hormones regulate expression of transcription factors and what role *LEC1* plays in *ssm1* small seed formation are still to be elucidated. To build up the interaction network of *LEC1*, *WRI1*, *AhPKp2*, and the plant hormones would provide useful information for us to understand the regulatory mechanism of peanut seed development.

## 4. Materials and Methods

### 4.1. Phenotypic Characteristics of SSM1 Mutant

The peanut mutant library was derived from cultivar Luahua 11 (LH11) through irradiation by 60Co γ-ray. In M2 generation of the library plants, a line with all of the seeds were smaller than LH11 and was screened, which in M3 generation, the small seed phenotypic can be stably inherited. After self-crossing for another two generations, the stable small seed peanut line was obtained and named small seed mutant 1 (*ssm1*). The peanut seeds were planted in Jiyang Experimental Farm, Shandong Academy of Agricultural Sciences. The pod and seed size and seed weight of *ssm1* and LH11 in the same development stage was compared. The seeds were dried in an oven and powdered in a pestle and mortar. The dry seed powder was used to determine the crude protein and oil content by using micro-Kjeldahl method and Soxhlet extraction method, respectively, according to the method described in previous studies [56,57].

The plump peanut seeds of *ssm1* and LH11 were selected for germination. Twenty seeds were germinated as one group, and five replicates were used in this study. The seeds were placed into 15 cm petri dish with a layer of filter paper on top and bottom, cultured in an incubator at 25 ± 2 °C under dark condition. The germination rate was calculated every 24 h, and the emergence of radicle was used as the germination standard. Germination potential = (number of germinated seeds at day 3/total number of tested seeds) × 100%; Germination rate = (number of final germinated seeds at day 7/total number of tested seeds) × 100%.

### 4.2. Paraffin Sectioning

The developing pods at DAP0 (0 day after gynophore penetrated the soil, gynophore above ground, not touching the soil), DAP3 (3 days after gynophore penetrated the soil), and DAP6 (6 days after gynophore penetrated the soil) were collected, respectively. The 0.5~1 cm gynophore tips were collected and approximately 20 gynophores from each stage were used for paraffin sectioning. Samples were fixed immediately in formalin-acetic acid-alcohol (FAA) for 24 h at 4 °C, then washed and dehydrated with a gradient ethanol series (70, 85, 95, and 100%). After dehydration, the tissues were cleared with xylol, embedded in paraffin, and sectioned into 8~10 μm sections. After drying at 37 °C, sections were de-paraffinized and hydrated in an ethanol gradient series (100, 95, 85, 70, 50, 30%, and distilled water) before being stained with toluidine blue O (TBO) reagent. After clearing and mounting, sections were observed under a microscope. Early pods (DAP10 and DAP20) were sliced freehand with double-sided blades and photographed under a stereoscope.

### 4.3. Determination of Endogenous Plant Hormones

The contents of IAA, cytokinin and ABA were measured with the seed tissues of DAP20 from *ssm1* and LH11. Approximately 1.0 g of each sample was rapidly frozen in liquid nitrogen and ground into a powder, then extracted with 1 mL methanol/water/formic acid (15:4:1, V/V/V). The quantification of endogenous phytohormones were conducted according to the manufacturer’s instructions (Wuhan Metware Biotechnology Co., Ltd., Wuhan, China) and using the external standard method. The sample extracts were analyzed using an LC-ESI-MS/MS system (UHPLC, ExionLC™ AD; MS, Applied Biosystems 6500 Triple Quadrupole). The analytical conditions were as follows, HPLC: column, Waters ACQUITY UPLC HSS T3 C18 (100 mm × 2.1 mm i.d., 1.8 µm); solvent system, water with 0.04% acetic acid (A), acetonitrile with 0.04% acetic acid (B); gradient program, started at 5% B (0–1 min), increased to 95% B (1–8 min), 95% B (8–9 min), finaly ramped back to 5% B (9.1–12 min); flow rate, 0.35 mL/min; temperature, 40 °C; injection volume: 2 μL. Three biological replications were performed.

### 4.4. RNA Extraction, RNA-Seq and Bioinformatic Analyses

The *ssm1* and LH11 immature seeds of DAP10 (10 days after gynophore penetrated the soil), DAP20 (20 days after gynophore penetrated the soil), and DAP40 (40 days after gynophore penetrated the soil) were collected for RNA extraction and sequencing. The samples were frozen immediately in liquid nitrogen and stored at −80 °C for RNA extraction using Trizol Reagent Kit (TaKaRa, Inc., Dalian, China). Three biological replicates were used for each sample. Total RNA was used to enrich mRNA with Oligo (dT) and sequenced using BGISEQ-500 platform (BGISEQ-500) at Beijing Genomics Institute (BGI; Shenzhen, China).

After removing the adaptor sequences and low-quality reads by SOAPunke and trimmomatic software, we obtained the clean reads. The sequences were uploaded to Short Read Archive of NCBI. The clean reads were aligned to peanut reference genome (https://www.peanutbase.org/data/public/Arachis_hypogaea/) using HISAT2 program and to reference gene by Bowtie2 [58,59]. The gene expression level was represented with FPKM (Fragments Per Kilobase per Million reads) calculated by RSEM method [60]. Relative gene expression level between *ssm1* and LH11 was represented by log2 ratio. The differentially expressed genes (DEGs) were identified using DEseq2 method with fold change ≥ 2 and Q-value (adjusted *p*-value) ≤ 0.05 [61].

Gene function was annotated by different databases including NCBI non-redundant protein sequences (Nr), gene ontology (GO), and Kyoto Encyclopedia of Genes and Genomes (KEGG) database. The GO and KEGG enrichment analyses were performed using R software and hypergeometric test. MeV software (version 4.9.0, The Institute for Genomic Research, Rockville, MA, USA, http://mev.tm4.org/) was used to generate the heatmaps.

### 4.5. Verification of RNA-Seq Data by qRT-PCR

qRT-PCR was used to validate the expression levels of selected genes. RNA samples were those used for RNA-seq, and the reverse transcription was performed using PrimeScript II 1st Strand cDNA Synthesis Kit (TaKaRa). The gene-specific primers were designed using PerlPrimer software (version 1.1.19, Owen Marshall, Parkville, Australia, http://perlprimer.sourceforge.net. Supplementary Table S6). qRT-PCR reaction was performed on ABI7500 Real Time System (Applied Biosystems) using TB Green™ Premix Ex Taq™ II (TaKaRa). The parameters of thermal cycle were 94 °C for 10 min, followed by 40 cycles of 94 °C for 15 s and 60 °C for 1 min. Three biological replications were performed for each reaction with actin gene as internal reference. The relative expression level of each gene between *ssm1* and LH11 was calculated by 2^−^^ΔΔCt^ method.

## 5. Conclusions

In the present study, a peanut small seed mutant *ssm1* was identified through irradiating peanut cultivar LH11 using 60Coγ ray. RNA-seq analysis identified DEGs involved in different pathways, a model for hormone signaling pathways and transcription factors cooperatively modulated seed development in *ssm1* was proposed, and the candidate genes leading to small seed size of the mutant was predicted. These results provided information for further understanding the mechanisms regulating seed size in peanut.

## Data Availability

The RNA-seq data in this study were available at NCBI Short Read Archive with the accession number of PRJNA841457 (https://www.ncbi.nlm.nih.gov/bioproject/PRJNA841457, accessed on 23 May 2022).

## Supplementary Materials

The following supporting information can be downloaded at: https://www.mdpi.com/article/10.3390/ijms23179726/s1.

## Abbreviations

DEGs, Differentially expressed genes; KEGG, Kyoto encyclopedia of genes and genomes; GO, Gene ontology; MAPK, Mitogen-activated protein kinase; Glc-6P, D-Glucose 1-phosphate; PEP, Phosphoenol pyruvate; TAG, Triglyceride; Phe, Phenylalanine; Trp, Tyrosine; MVA, Mevalonic acid; DMAPP, Dimethylallyl diphosphate; GGPP, Geranylgeranyl diphosphate; IAA, Indoleacetic acid; BRs, Brassinolides; CKs, Cytokinins ; ABA, Abscisic acid; GAs, Gibberellic acidS; PKp2, Plastidial pyruvate kinase 2; TAA, Tryptophan aminotransferase; YUC, Indole-3-pyruvate monooxygenase YUCCA; AMI, Amidase; ALDH, Aldehyde dehydrogenase; TIR1, Transport inhibitor response 1; ARF, Auxin response factor; SAUR, Small auxin up RNA; DWF4, 22a-hydroxylase/Cytochrome P450 90B1; CPD, Constitutive photomorphogenic dwarf/Cytochrome P450 90A1; CYP90C1, Cytochrome P450 90C1; 3-epi-6-deoxocathasterone 23-monooxygenase; Dwarf, cytochrome P450 85A; BAK1, BRI1-associated receptor kinase 1; BZR1, Brassinazole resistant 1; CYP735A, cytokinin hydroxylase/Cytochrome P450 735A; CKX, cytokinin dehydrogenase; CRE1, Cytokinin response 1; AHP, Histidine phosphotransfer proteins; ARR, A-type response Regulator; BCH1, beta-Carotene 3-hydroxylase 1; NCED, 9-cis-Epoxycarotenoid dioxygenase; ABA2, ABA deficient 2; ABA3, ABA deficient 3; ABI5, Abscisic acid-insensitive 5; PYL, Pyrabactin resistance 1-Llke; KAO, Ent-kaurenoic acid oxidase; KO, Ent-kaurene oxidase; GA20ox, GA 20-oxidases; GA2ox, GA 2-oxidases; GID1, GA-insensitive dwarf 1; DELLA, Proteins containing conserved DELLA domains in the N terminus; LEC1, Leafy cotyledon 1; WRI1, WRINKLED 1; ABI3, Abscisic acid-insensitive 3; FUS3, FUSC A3; RAV1, Related to ABI3/VP1 1; IKU2, HAIKU2; AP2, APETALA 2.
